# Role of *Candida* species from HIV infected children in enamel caries lesions: an *in vitro* study

**DOI:** 10.1590/1678-77572016-0021

**Published:** 2017

**Authors:** Senda CHARONE, Maristela Barbosa PORTELA, Karol de Oliveira MARTINS, Rosangela Maria SOARES, Gloria Fernanda CASTRO

**Affiliations:** 1 Universidade Federal do Rio de Janeiro, Faculdade de Odontologia, Departamento de Odontopediatria e Ortodontia, Rio de Janeiro, RJ, Brasil.; 2 Universidade de Brasília, Faculdade de Odontologia, Departamento de Cariologia, Brasília, DF, Brasil.; 3 Universidade Federal Fluminense, Faculdade de Odontologia, Departamento de Clínica e Odontopediatria, Niterói, RJ, Brasil.; 4 Universidade Federal do Rio de Janeiro, Instituto de Microbiologia Paulo de Góes, Departamento de Microbiologia, Rio de Janeiro, RJ, Brasil.

**Keywords:** Candida albicans, Dental caries, HIV infections, Child, Hardness

## Abstract

**Objectives:**

This study analyzed the capacity of *Candida* spp. from dental biofilm of HIV infected (HIV+) children to demineralize primary molar enamel in vitro by Transversal Microhardness (TMH), Polarized Light Microscopy (PLM) and the quantity of calcium ions (Ca^2+^) released from the enamel.

**Material and Methods:**

*Candida* spp. samples were isolated from the supragingival biofilm of HIV+ children. A hundred and forty (140) enamel blocks were randomly assigned to six groups: biofilm formed by *C. albicans* (Group 1); mixed biofilm formed by *C. albicans* and *C. tropicalis* (Group 2); mixed biofilm formed by *C. albicans* and *C. parapsilosis* (Group 3); mixed biofilm formed by *C. albicans*, *C. parapsilosis* and *C. glabrata* (Group 4); biofilm formed by *C. albicans* ATCC (Group 5) and medium without *Candida* (Group 6). Enamel blocks from each group were removed on days 3, 5, 8 and 15 after biofilm formation to evaluate the TMH and images of enamel were analyzed by PLM. The quantity of Ca^2+^ released, from Groups 1 and 6, was determined using an Atomic Absorption Spectrophotometer. The SPSS program was used for statistical analysis and the significance level was 5%.

**Results:**

TMH showed a gradual reduction in enamel hardness (p<0.05) from the 1^st^ to 15^th^ day, but mainly five days after biofilm formation in all groups. The PLM showed superficial lesions indicating an increase in porosity. *C. albicans* caused the release of Ca^2+^ into suspension during biofilm formation.

**Conclusion:**

*Candida* species from dental biofilm of HIV+ children can cause demineralization of primary enamel *in vitro*.

## Introduction

The most common oral lesion of HIV infected children is candidiasis^25^ and the major etiologic agent of this oral lesion is *C. albicans* ^26^. However, various studies have evidenced the presence of other species with pathogenic features such as *C. tropicalis*, *C. parapsilosis*, *C. glabrata*, *C. guillermondii* and *C. dubliniensis* ^22,24^. Studies in the literature have associated the presence of *Candida spp.* in the oral cavity with the development of caries lesions^7,30^. Nikawa, et al.^15^ (1994) suggested the possibility that biofilms colonized by *C. albicans* have increased cariogenicity, because this yeast can produce lactic acid through the fermentation of carbohydrates and degenerate the dental hydroxyapatite structure. Also, *C. albicans* can produce enzymes with collagenolytic activity (aspartic proteases) and dental collagen hydrolysate^17^.

Children infected with HIV tend to present a higher prevalence of caries compared with noninfected children^7,19^. The most probable hypotheses that support this are: the ingestion of a hypercaloric diet, a high sucrose content in medicines^20^, low immunosuppression^4^ and unsatisfactory oral hygiene^23^. Considering these possible roles of *Candida* in the development of caries and the fact that HIV infected children present a high prevalence of these fungi and caries lesions, further studies are needed. Also, other possible factors involved in the beginning and development of caries disease in these patients should be investigated.

In this study, we hypothesized that, *in vitro*, *Candida* spp. isolated from the dental biofilm of HIV infected (HIV+) children is able to demineralize primary molar enamel. Thus, strains of *C. albicans* and non*-albicans* from the dental biofilm of HIV+ children were isolated and identified. The *in vitro* capacity of these *Candida* spp. samples to demineralize primary molar enamel was analyzed by Transversal Microhardness (TMH) and Polarized Light Microscopy (PLM) and furthermore the quantity of calcium ion (Ca^2^) released from the enamel was determined.

## Material and methods

### Microorganisms

The *Candida* spp. samples were isolated from the thick supragingival biofilm^23^ on the dental surfaces of fifty (50) patients of both genders, aged between 3 and 12 years old with definitive diagnosis for HIV infection according to the CDC criteria^6^. The HIV+ children were selected by convenience over an 8-month period. These children attended the Pediatric AIDS Outpatient Clinic at the Pediatric Hospital of the Federal University of Rio de Janeiro (IPPMG-UFRJ) on a regular basis and were assisted by the School of Dentistry of the same institution. Children who had been under antifungal therapy in the least three months or used topic oropharyngeal antimicrobial drugs were not included in the study. Other medical data were obtained from their medical records.

To collect the biofilm, standard dental curettes were rubbed against the easiest accessed dental surface that allowed relative isolation of saliva. The material collected was transferred to Eppendorffs tubes containing 1 mL of NaCl–0.85%, and then kept under refrigeration until analysis. For the analysis, 100 mL of these suspensions was seeded onto Petri plates containing CHROMagar *Candida* ^®^ medium and incubated at 37°C for 72 hours^18^. Colonies were analyzed by using biochemical tests of fermentation and sugar assimilation (API 20C System, Biomerieux, Marcy L’Étoile, Lyon, France). Also, green colonies were inoculated in Sabouraud dextrose agar to screen their ability to grow at 45°C in 48 hours and thus differentiate between *C. albicans* and *C. dubliniensis* ^8^ *.*

This study was approved by the Ethics Committee of the UFRJ and informed consent form was obtained from the caregivers of the children.

### Preparation of specimens of human primary teeth

Forty (40) sound primary molars without any visible alteration (stereoscopic microscope, 40x, Astro Optics Division, Montpelier, Vermont, USA) were selected. They were cut by a double sided diamond disc, forming 140 enamel blocks (5x5x2 mm). After cleaning^5^ all the areas were protected by nail varnish (two layers, with 24 hours for drying each layer) except for a circular area with 6.25 mm^2^in the center of each block. This area was exposed to *Candida* biofilms, while the protected area served as its own control since it was not exposed to the biofilm.

### Biofilm formation

After quantification and identification of *Candida spp.,* we observed that 45 patients presented positive growth of only *C. albicans* in the supragingival biofilm; three presented positive growth of *C. albicans* and *C. parapsilosis* simultaneously*;* one, positive growth of both *C. albicans* and *C. tropicalis*; and one, positive growth of *C. albicans*, *C. parapsilosis* and *C. glabrata* simultaneously*.* Thus, five (5) strains of *C. albicans* were randomly selected from five (5) different patients, who had just presented this species. Also, isolates of *C. albicans* and non-*albicans* pertaining to patients who simultaneously presented isolates of: *C. albicans + C. parapsilosis; C. albicans + C. tropicalis;* and *C. albicans + C. parapsilosis + C. glabrata* (one isolate of each species) were selected. One reference isolate of *C. albicans* (ATCC 24433) was used.

The 140 blocks were sterilized^1^ and fixed in 24-well plates containing YCB-agar culture medium (Yeast Carbon Base, Difco, Trenton, New Jersey, USA), with 1% BSA (Bovine Serum Albumin)^8^. Ten 24-well plates containing 14 dental blocks each were divided into six groups: Group 1 – enamel exposed to *C. albicans* biofilm (n=70 blocks; five plates)*;* Group 2 – mixed biofilm formed by *C. albicans* and *C. tropicalis* (n=14; one plate)*;* Group 3 – mixed biofilm formed by *C. albicans* and *C. parapsilosis* (n=14; one plate)*;* Group 4 – mixed biofilm formed by *C. albicans, C. parapsilosis* and *C. glabrata* (n=14; one plate)*;* Group 5 – *C. albicans* biofilm (ATCC 24433) (n=14; one plate); Group 6 (control group) – absence of *Candida* biofilm (n=14; one plate). Standard cell suspensions containing 10^5^yeasts/mL sowed in YCB medium supplemented with BSA 1% under mixing for 48 hours at 37°C were inoculated in each well, except for the control. The biofilm formation occurred after incubation at 37°C.

Two (2) dental blocks were removed from each plate after the 1^st^, 3^rd^, 5^th^, 8^th^, 10^th^, 12^th^ and 15^th^ day, and inserted in Falcon tubes containing 5 mL of NaCl (0.85%) and vortexed for one minute. Biofilm cell viability was assessed through the Trypan blue exclusion test. All blocks were sterilized, cleaned using a soft brush with water and pumice paste and then stored in a pot containing cotton soaked in NaCl for future analysis.

### Transversal Microhardness (TMH)

The enamel TMH was measured using a Knoop indenter with a 50 gram load for 15 seconds^8,10^. The blocks were longitudinally sectioned through the equatorial region by a precision sectioning cutter (Isomet, Buehler, Lake Bluff, Illinois, USA) with a double sided diamond disc, forming two enamel blocks. Only one half of each block was built-in acrylic resin and its enamel surface was polished with mesh sandpaper (1000 grit, 1200 grit and 2400 grit) (3M, Sumaré, São Paulo, Brazil) for 10 minutes. Then, a polishing machine with felt discs (Arotec Ind. & Com. Ltda; São Paulo, São Paulo, Brazil) and abrasive alumina (1 and 0.3 µm; South Bay Technology Inc.; San Clemente, California, USA) were used until the surfaces were plain and smooth. Indentations were made at distances of 12.5, 25, 37.5, 50, 62.5, 75, 87.5, 100, 112.5, 125, 137.5, 150 µm from the external surface into the dentin-enamel junction (DEJ)^28^. All the previously patterned indentations had been made in the enamel areas exposed to the biofilm as well as in those protected areas.

### Polarized Light Microscopy (PLM) analysis

For the qualitative analysis, 14 enamel fragments remaining from the blocks used in Group 1 were selected. They were examined through a 100x magnification PLM under crossed Nichols, with quartz accessories. The fragments were prepared by hand using increasing grades of water sandpaper (1000 grit, 1200 grit and 2400 grit). After that, they were polished with felt polishing paste until a 100 µm longitudinal section was observed. This measure was obtained by using a digital micrometer.

### Determination of Ca2+ released

To determine the amount of calcium ion (Ca^2^) released from the blocks, an Atomic Absorption Spectrophotometer (air flame/nitrous oxide) (ContrAA 300, Analytik Jena, Uberlingen, Germany) equipped with a calcium-specific hollow cathode lamp was used at the following operating conditions: lamp current 3 mA; fuel, acetylene/nitrous oxide flame; wavelength, 423 nm. Briefly, 1 mL of the suspension from one plate (selected randomly) of Group 1 (*C. albicans,* n=14) and from the plate of Group 6 (control group, n=14) was collected from each well on the 1^st^, 3^rd^, 5^th^, 8^th^, 10^th^, 12^th^ and 15^th^ day. After centrifugation (3000 xg, for 3 minutes, at 4°C) the supernatant portions were stored in plastic tubes with screw caps containing 250 µL of nitric acid 65% (HNO_3_) and kept under refrigeration until analysis. For the analysis, 200 μL of the sample was put into 3 mL of 53 mM La (NO_3_)_3_ and then 50 mM HCl was added^27^. Standard solutions containing 10, 20, 40 and 80-μg/mL calcium were prepared. The readings of the calcium ion release were compared with a standard curve obtained from readings of the standard solutions. Hence, the calcium release was calculated on the 1^st^, 3^rd^, 5^th^, 8^th^, 10^th^, 12^th^ and 15^th^ day.

### Statistical analysis

In order to determine the TMH, the sum of the indentation values was made, followed by the arithmetic mean. Data were analyzed by SPSS Statistics Program 20. Parametric statistical tests were used for correlations and the significance level was 5% (p<0.05). The means of TMH, on the different days, in each group, were compared using the Analysis of Variance (ANOVA). Comparisons between the TMH values of the exposed and nonexposed areas were made for Group 1 using the T-Student test. For the other groups, only a descriptive analysis was made due to the small sample size. Also, the results of PLM and Ca^2^ releases were descriptively analyzed.

## Results

For the *in vitro* experiments, from the 45 patients that presented positive growth only for *C. albicans* in the supragingival biofilm, five (5) *C albicans* isolates were randomly selected from five different patients, resulting in a sample of 70 dental blocks for Group 1 (14 blocks for each isolate). However, the number of patients that presented positive growth of *C. albicans* and other species simultaneously were very few. Thus, one isolate of *C. albicans* and one of non-*albicans,* pertaining to patients who simultaneously presented these isolates, were selected [Group 2: *C. albicans + C. parapsilosis;* Group 3: *C. albicans + C. tropicalis;* Group 4: *C. albicans + C. parapsilosis + C. glabrata* (one isolate of each specie)], resulting in 14 dental blocks for each group.

The results of TMH are shown in Table 1. A gradual decrease (p<0.05; ANOVA) of the TMH values in areas exposed to biofilm was observed over the period (from the 1^st^ to 15^th^ day) in all groups exposed to *Candida* biofilm (Groups 1 to 5). In nonexposed areas this variation was not significant for these groups. In Group 6 (no *Candida* biofilm formation) both exposed and nonexposed areas did not present any significant difference in TMH values.

**Table 1:** Mean values of transversal microhardness (TMH) of primary enamel according to the groups and duration (in days) of the experiment

	Group 1 (n=70) Mean±SD		Group 2 (n=14) Mean±SD		Group 3 (n=14) Mean±SD		Group 4 (n=14) Mean±SD		Group 5 (n=14) Mean±SD		Group 6 (n=14) Mean±SD	
Day	Nonexposed enamel	Exposed enamel	Nonexposed enamel	Exposed enamel	Nonexposed enamel	Exposed enamel	Nonexposed enamel	Exposed enamel	Nonexposed enamel	Exposed enamel	Nonexposed enamel	Exposed enamel
1^st^	311.4±12.2	317.7±19.4	324.5±6.9	321.0±10.8	320.8±1.1	336.9±3.6	330.5±8.2	344.1±14.7	327.2±12.9	326.1±12.0	334.0±4.9	345.2±36.0
3^rd^	305.6±17.8	302.5±18.7	310.7±20.0	315.4±25.7	335.7±1.07	350.1±34.2	322.9±2.4	314.7±13.9	314.6±5.3	317.8±4.8	327.0±25.8	330.4±23.1
5^th^	304.7±9.4^A^	284.8±28.7^A^	307.9±13.2	245.9±13.46	332.6±1.2	287.9±15.3	335.5±5.7	255.29±7.8	327.1±10.0	323.8±12.2	325.6±6.5	338.5±1.47
8^th^	302.3±8.9^B^	199.3±29.0^B^	328.1±4.5	241.0±32.3	318.7±6.2	240.0±1.5	324.9±17.9	249.9±23.2	337.0±19.0	329.7±15.3	307.1±3.02	337.8±1.99
10^th^	301.9±10.8^C^	173.3±20.3^C^	318.7±8.1	233.3±26.3	322.9±3.3	235.82±7.2	323.8±8.9	234.8±11.6	334.6±1.8	231.3±13.9	309.0±4.6	332.6±3.92
12^th^	296.1±12.5^D^	153.9±20.1^D^	336.2±12.2	214.7±1.8	335.0±20.0	223.6±2.6	327.8±2.1	212.9±11.53	331.1±10.9	238.0±3.0	310.4±10.2	333.6±13.1
15^th^	300.0±14.9^E^	145.6±13.6^E^	339.7±1.3	210.5±13.1	326.5±4.0	221.5±6.4	316.2±17.5	206.4±12.2	326.8±7.3	227.0±18.1	307.9±7.6	319.0±20.0
ANOVA* *p* value	0.218	0.000	0.141	0.003	0.315	0.000	0.698	0.000	0.553	0.000	0.214	0.868

Notes: 1) Similar letters indicate statistical differences (comparison per line) between the exposed enamel and the nonexposed enamel for the same period (Student's t-test); A p=0.04; B p=0.0002; C p=0.0002; D p=0.0002; E p=0.0002

2) * ANOVA p value indicates the comparison beteween different periods of exposure in the same enamel (comparison per column)

3) Group1: biofilm formed by *C. albicans*; Group 2: Mixed biofilm formed by *C. albicans* and *C. tropicalis*; Group 3: Mixed biofilm formed by *C. albicans* and *C.parapsilosis*; Group 4: Mixed biofilm formed by *C. albicans*, *C. parapsilosis* and *C. glabrata*; Group 5: biofilm formed by *C. albicans* (ATCC 24433); Group 6: absence of biofilm (control group)

The TMH values of exposed and nonexposed areas were daily compared only in the group with *C. albicans* biofilm (Group 1). There was no difference on the 1^st^ and 3^rd^day, but from the 5^th^ day on, a significant difference (p<0.05; Student’s t-test) was observed. In groups with mixed biofilm (Groups 2, 3 and 4), the results show a similar behavior in Groups 2, 3 and 4. In Group 5 the decrease in the TMH values may be observed as of the 10^th^ day, however, in Group 6, TMH values were similar throughout the experiment (Table 1). However, statistical test for these comparisons in groups with mixed biofilm were not carried out because of the reduced sample size in each day, in these groups.

**Figure 1:**
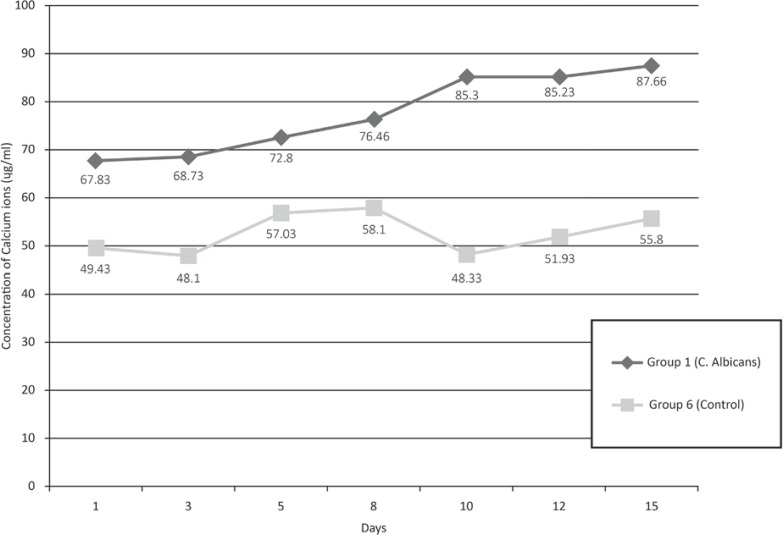
Polarized light microscopy (PLM) images of the enamel blocks from Group 1 (*C. albicans* biofilm), according to the different periods of exposure. Images A, C, E and G are the areas of enamel exposed to biofilm of *C. albicans* on 1st, 5th, 8th and 15th day respectively. Images B, D, F and H represent the respective controls (areas not exposed to biofilm)

Mineral losses observed using PLM showed that enamel areas exposed to *C. albicans* biofilm presented a superficial zone with negative birefringence (double refraction) and lesions in the sub-surface zone with positive birefringence (double refraction), indicating an increase in porosity. This porosity increase along the enamel-dentin junction, increased during the experiment. In Figure 1, the images 2A, 2C, 2E and 2G show the enamel areas exposed to *C. albicans* during the 1^st^, 5^th^, 8^th^ and 15^th^ days respectively. The images 2B, 2D, 2F and 2H show their respective controls (protected areas).

**Figure 2:**
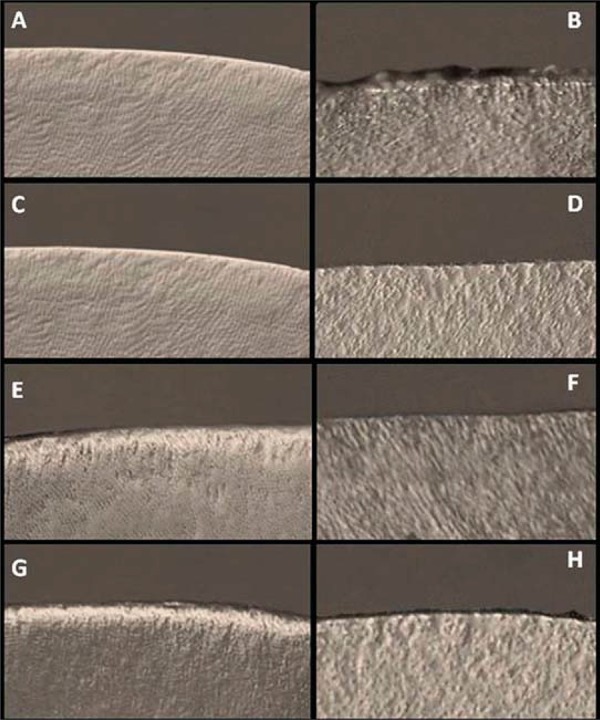
Release of calcium ions (µg/mL) during *C. albicans* biofilm formation, according to the different periods of exposure. A=control, B=1st day, C=3rd day, D=5th day, E=8th day, F=10th day, G=12th day and H=15th day

The quantity of Ca^2^ ions released into the medium during the *Candida albicans* biofilm forming experiment is shown in Figure 2. There was an increase in the quantity of Ca^2^ ions released over the days of the experiment in Group 1 (*C. albicans* biofilm).

## Discussion

Nikawa, et al.^16^ (2003) observed that *C. albicans* presents cariogenic potential and has the ability to dissolve hydroxyapatite crystals in large proportions. According to these authors, this species has collagenolytic activity, sticking to the collagen in the regular and denatured forms by different mechanisms, which could contribute to the persistence of the yeast on the dissolved hydroxyapatite surface^14,21^. Our results show that *Candida* spp., in mixed biofilm or not, has a potential to cause enamel demineralization *in vitro*, since the microhardness analysis of enamel showed a significant decrease along the experiment. Also, we noted that in the *C. albicans* biofilm group, from the 5^th^ day on, the TMH values of exposed and nonexposed areas were different. We hypothesized that this may have been due to the fact that yeasts present a more intense metabolism after the 5^th^ day or this result could be associated with an increase of lactic acid production along the days, as an accumulative effect. In addition, a recent study from our group showed that mature biofilm of *C. albicans* can be observed as of the 5^th^ day, under the same experimental conditions (data not shown).

Secreted aspartyl proteinases (Saps) are among the most important virulence factors of *Candida* spp. Their relationship to the development of candidiasis by adhesion to human tissues, degradation of extracellular matrix and other important proteins associated with human defense is well known. Recently, Li, et al.^13^ (2014) demonstrated the role of *Candida albicans*-Saps in severe early childhood caries. The authors observed by enzymatic activity that the species isolated from the biofilm of children with severe early childhood caries were statically higher producers of Saps than the *C. albicans* isolates of caries free group. Moreover, different members of the Sap family might be differentially expressed depending on the environment and host conditions. The gene expression levels of Sap1–5 are the predominant protease genes expressed in *C. albicans* from dental biofilm and Sap1 may play an important role in the development of severe early childhood caries^13^. Additionally, Brighenti, et al.^3^ (2014) also observed caries associated with other virulence factors (production of acid, extracellular polysaccharides, proteins and metabolic activity) of *C. albicans* biofilm isolated from the saliva of patients with sickle-cell anemia. These authors demonstrated that *C. albicans* biofilms from patients with sickle-cell anemia presented a greater caries-associated virulence than isolates from healthy children.

A limitation of our study was the small sample size of the groups exposed to mixed biofilm formed by *C. albicans* and non*-albicans*, which did not allow a quantitative analysis between TMH values of the exposed versus nonexposed areas. Therefore, these results must be discussed with caution. On the other hand, our descriptive analysis shows that the values of the TMH of blocks exposed to a mixed biofilm formed by *C. albicans* and non-*albicans* species presented a profile similar to those exposed only to *C. albicans*, but with a smaller decrease. Some competition between the *Candida* species may be occurring in the mixed biofilm that could decrease the activity of the *C. albicans* species in the system^29^. Future investigations of the demineralization profile of each *Candida* non-*albicans* isolated could help to explain this phenomenon of a reduced demineralization in a mixed biofilm.

On the other hand, although some results were descriptively analyzed, the comparability of our results with those in the literature shows some consistency^8,21,30^. Although this *in vitro* study shows a confirmatory result, few studies in the literature show the *in vitro* demineralization potential of *Candida* spp. biofilm and the real role of these yeasts in caries disease processes. Besides, there are studies that have already demonstrated a potential synergism among *Candida albicans* and *Streptococcus mutans* ^9,12^ *.* However, we did not consider *S. mutans* in our experiments in order to only compare the demineralization potentials of *Candida albicans* and non-*albicans*, since our objective is to understand the real demineralization profile of these yeasts. Additionally, the importance of *S. mutans* in the development of caries lesions is already known in the current literature. However, novel investigations regarding the synergistic demineralization potential of a mixed biofilm of *Candida* spp., isolated from oral cavity of HIV infected children, and *S. mutans* are been initiated to better understand the biological significance.

The small variation of microhardness values on the enamel nonexposed to *Candida* spp. biofilm (Groups 1 to 5), as well as the whole of Group 6 (control group), shows that the pH variation of the culture medium was not able to cause any significant mineral loss. Moreover, our results showing the loss of calcium ions by atomic force absorption and the polarized light images also reflected the changes found in microhardness of the enamel exposed to *C albicans* biofilm over the days of the experiment. However, on some days, in the control group (Group 6), the calcium concentration in the suspension reduced instead of increasing gradually. This could be explained by the fact that the quantification of calcium release was performed in only one isolate *per* day, randomly selected, and the isolate used may have been one of those that causes less mineral loss.

Although various studies have already shown the demineralization ability of *Candida* species isolated from other oral cavity sites^11,30^, the literature is very scarce in studies about strains from HIV-infected children, who have high caries prevalence. In parallel, these yeasts have characteristics that are strain dependent principally when participating in a biofilm environment. Arzmi, et al.^2^ (2015) showed that different strains of *C. albicans* present a distinct profile of coaggregation with *A. naeslundii* and *S. mutans* in a biofilm environment. These data reinforce the importance of this study.

## Conclusions

1. Our study confirms the capacity of *Candida* spp. isolates from dental biofilm of HIV infected children to cause *in vitro* enamel demineralization.

2. Confirming the cariogenic potential of yeasts and elucidating the exact participation of *Candida* spp. in the development of caries will contribute to caries disease control in HIV infected patients.
